# Alcohol Not a Stimulant

**Published:** 1891-11

**Authors:** 


F. E. DANIEL, M. D., EDITOR AND PUBLISHER.

THIS JOURNAL, by unanimous vote of the members, was made the Official Organ of the Austin District Medical Society.

COLLABORATORS:

*Dr. R. M. Swearingen, Austin.*
*Prof. B. E. Hadra, M. D., Galveston.*
*Prof. Geo. Cupples, M. D., San Antonio.*
*Dr. T. C. Osborn, Cleburne, Texas.*
*Dr E. J. Doering, Chicago.*
*Dr. E. J. Beall, Fort Worth.*
*Dr Odo Betz, Germany.*
*Prof. W. B. Rogers, M. D., Memphis.*
*Dr. J. W. McLaughlin, Austin.*
*Dr. T. J. Tyner, Austin.*
*Prof. J. F. Y. Paine, M. D., Galveston.*
*Dr. R. H. L. Bibb, Mexico.*
*Dr. T. J. Bennett, Austin.*
*Dr. Bat Smith, Wharton.*
*Dr. E. Meierhof, New York.*
*L. H. Luce, M. D., West Tisbury, Mass.*

### ALCOHOL NOT A STIMULANT.

It will doubtless be news to many, and will by many be denied, that alcohol is not a stimulant! For thousands of years it has been a popular belief, firmly rooted in the mind, that alcohol in the shape of whisky is *the* stimulant *par excellence*, and under this delusion it has worked untold injury; in the sick room more especially. There are many who to-day would as soon believe that the sun does not shine as to believe that alcohol does not stimulate.

That it causes momentary excitement cannot be denied; but it appears from the best evidence to be had that it is, in reality, the very reverse of stimulant,—a depressant. There is no subject connected with medicine which has undergone a more complete revolution than that of alcohol in disease. For many years it was claimed to be a food;—it was claimed that it was conservative of tissue, etc., and indeed convincing proofs have been brought forward from time to time to clinch it. It is related by some English authority that an old man had been found who for twenty years had taken no food except a very small piece of bread daily, and had lived on gin. All during the civil war whisky—such as we could get, and frequently the meanest sort, was the sheet anchor in typhoid fever and pneumonia. That it was abused there is no doubt; that many were actually killed by it, is equally certain. That it was beneficial in some cases also cannot be disputed.

To some extent, or in some sense, alcohol may be said to be a food, or to take the place of food, *i. e.*, by its action in diminishing vaso-motor and excito-motor nerve force it diminishes or retards tissue metabolism and excretory products, combustion and waste; in other words, by diminishing waste of tissue, the demand for supply is also lessened. It is the testimony of abundant clinical experiences that a person can maintain a given standard of weight on a diminished amount of food, if a portion of alcohol be allowed daily.

There is also no subject in medicine on which there is a wider difference of opinion—or we may say, a greater diversity of opinions than on this. But, we will confine our remarks to America, and we think we are safe in saying that amongst American physicians there has been a revolution—it is still going on—on the subject, and will cite as authority the eminent Prof. N. S. Davis. Dr. Davis is regarded as authority on therapeutics, and he says (address at Prohibition Park, 1891), most emphatically, that alcohol is, in no sense, a food, either directly or indirectly; he declares also, as the result of his clinical observation extending over a period of fifty years, that alcohol is not a stimulant; and is not even a tonic. On the contrary, he says its effects are *always* depressing, paralysing and poisonous. He strongly advises physicians to abandon its use in practice, and holds that "there are no proper or necessary uses for alcohol as a medicine."

Every physician of experience must have observed that in overdoses alcohol paralyses the pneumogastric nerve, and retards breathing in as marked manner as does opium, and that acute alcoholic poisoning very closely resembles opium narcosis. Respiration is diminished to that extent that the blood is not oxydised and the patient becomes livid, and finally dies, apparently of asphyxia. We remember once seeing four learned doctors in attendance on a brother doctor who was suffering from pneumonia. They had given him whisky till, in our opinion he was suffering frsm its effects; was blue in the face, with slow labored and stertorous respiration, lips swollen, and extremities cold and covered with a clammy perspiration. The lungs were laboring in vain to throw it off; but at the next consultation it was decided that he "needed stimulating," and the amount of whisky was doubled. The funeral was largely atttended.

But how strange it sounds that the world's acknowledged stimulant—par excellence—the stimulant that for ages has stood at the head of its class, is not a stimulant! When will wonders cease? Is "nothing true but heaven?" When we were a boy we got hold of a book called "Curious myths of the middle ages," and in it we read that William Tell was a myth; that no such person ever existed. . We were grieved that an "idol" so long enshrined in our youthful mind should be thus at one fell stroke dashed to pieces. So it will be to many a "stimulaut" doctor on reading this "news," and to many an old toper who firmly believes that but for his customary "stimulant" he would have long since been in his grave. What are we to believe, if it can be proved at this late day that the typical stimulant of many ages is the reverse of a stimulant; that old king alcohol has been masquerading all these years, a veritable wolf in sheep's clothing?

